# Identification of compound heterozygous variants in the noncoding *RNU4ATAC* gene in a Chinese family with two successive foetuses with severe microcephaly

**DOI:** 10.1186/s40246-018-0135-9

**Published:** 2018-01-25

**Authors:** Ye Wang, Xueli Wu, Liu Du, Ju Zheng, Songqing Deng, Xin Bi, Qiuyan Chen, Hongning Xie, Claude Férec, David N. Cooper, Yanmin Luo, Qun Fang, Jian-Min Chen

**Affiliations:** 1 Fetal Medicine Centre, Department of Obstetrics and Gynaecology, The First Affiliated Hospital of Sun Yat-Sen University, Guangzhou, China; 2 Department of Dermatology, Guangzhou Institute of Dermatology, Guangzhou, China; 3 Department of Ultrasonic Medicine, The First Affiliated Hospital of Sun Yat-Sen University, Guangzhou, China; 4 Guangzhou KingMed Center for Clinical Laboratory, Guangzhou, China; 5 Dongguan Women and Children’s Hospital, Dongguan, China; 6 UMR1078 “Génétique, Génomique Fonctionnelle et Biotechnologies”, INSERM, EFS - Bretagne, Université de Brest, CHRU Brest, Brest, France; 7 Institute of Medical Genetics, School of Medicine, Cardiff University, Cardiff, UK; 8 INSERM UMR1078, EFS, UBO, 22 avenue Camille Desmoulins, 29238 Brest, France

**Keywords:** Genetic counselling, Microcephalic osteodysplastic primordial dwarfism type 1, MOPD1, Noncoding *RNU4ATAC* gene, Prenatal diagnosis, RNA secondary structure, Small nuclear RNA, Taybi-Linder syndrome, WES, Whole-exome sequencing

## Abstract

**Background:**

Whole-exome sequencing (WES) over the last few years has been increasingly employed for clinical diagnosis. However, one *caveat* with its use is that it inevitably fails to detect disease-causative variants that occur within noncoding RNA genes. Our experience in identifying pathogenic variants in the noncoding *RNU4ATAC* gene, in a Chinese family where two successive foetuses had been affected by severe microcephaly, is a case in point. These foetuses exhibited remarkably similar phenotypes in terms of their microcephaly and brain abnormalities; however, the paucity of other characteristic phenotypic features had made a precise diagnosis impossible. Given that no external causative factors had been reported/identified during the pregnancies, we sought a genetic cause for the phenotype in the proband, the second affected foetus.

**Results:**

A search for chromosomal abnormalities and pathogenic copy number variants proved negative. WES was also negative. These initial failures prompted us to consider the potential role of *RNU4ATAC*, a noncoding gene implicated in microcephalic osteodysplastic primordial dwarfism type-1 (MOPD1), a severe autosomal recessive disease characterised by dwarfism, severe microcephaly and neurological abnormalities. Subsequent targeted sequencing of *RNU4ATAC* resulted in the identification of compound heterozygous variants, one being the most frequently reported MOPD1-causative mutation (51G>A), whereas the other was a novel 29T>A variant. Four distinct lines of evidence (allele frequency in normal populations, evolutionary conservation of the affected nucleotide, occurrence within a known mutational hotspot for MOPD1-causative variants and predicted effect on RNA secondary structure) allowed us to conclude that 29T>A is a new causative variant for MOPD1.

**Conclusions:**

Our findings highlight the limitations of WES in failing to detect variants within noncoding RNA genes and provide support for a role for whole-genome sequencing as a first-tier genetic test in paediatric medicine. Additionally, the identification of a novel *RNU4ATAC* variant within the mutational hotspot for MOPD1-causative variants further strengthens the critical role of the 5′ stem-loop structure of U4atac in health and disease. Finally, this analysis enabled us to provide prenatal diagnosis and genetic counselling for the mother’s third pregnancy, the first report of its kind in the context of inherited *RNU4ATAC* variants.

## Background

Microcephaly is usually defined in terms of a head circumference more than two standard deviations below the mean for age and sex; it can occur in the womb or may develop during the first few years of life [1, 2]. Abnormal growth of the head may occur as a consequence of a number of factors, both genetic and environmental (e.g. exposure to certain viruses such as rubella, drugs and alcohol during pregnancy) [3]. The genetic causes are highly heterogeneous; thus, a search for microcephaly in the Human Phenotype Ontology database [4] yielded 652 genes. Depending on the precise nature of the condition involved, microcephaly may be associated with seizures, developmental delay, intellectual disability or other problems. It may even be associated with substantial physical disability and premature death; there is no treatment for microcephaly. Therefore, it is extremely important to identify the genetic causes of severe microcephaly in affected families with a view to providing prenatal diagnosis and genetic counselling in subsequent pregnancies.

With the decreased cost of next-generation sequencing, whole-exome sequencing (WES) has rapidly evolved from its original application as a tool for gene discovery in research settings to an important diagnostic tool in a clinical context [5–7], especially for diseases that are characterised by a significant level of genetic heterogeneity [8]. However, one *caveat* with WES is that disease-causative variants which occur within noncoding RNA genes will invariably be missed [9, 10]. Here, we highlight this issue by describing our experience of identifying novel compound heterozygous variants in the noncoding *RNU4ATAC* gene (OMIM #601428), in a Chinese family with two successive foetuses affected by severe microcephaly.

## Results

### Family description

A 30-year-old woman was referred to our centre at the First Affiliated Hospital of Sun Yat-Sen University after her second foetus (II:2) had been found to have severe microcephaly at 24 gestational weeks (GW), just as her first one (II:1; Fig. 1a) had previously. Clinical findings in the two affected foetuses, who were terminated at 36 GW (II:1) and 30 GW (II:2) respectively, are illustrated in Fig. 2 and summarised in Table 1. However, no precise diagnosis of the underlying abnormality could be made based upon these clinical findings owing to the paucity of characteristic features beyond severe microcephaly. In the case of II:1, standard G-banding karyotyping using cord blood cells taken at 35 GW revealed a normal karyotype, whereas chromosomal microarray analysis failed to detect any pathogenic copy number variations; no further analyses were performed at the time.

**Fig. 1:**
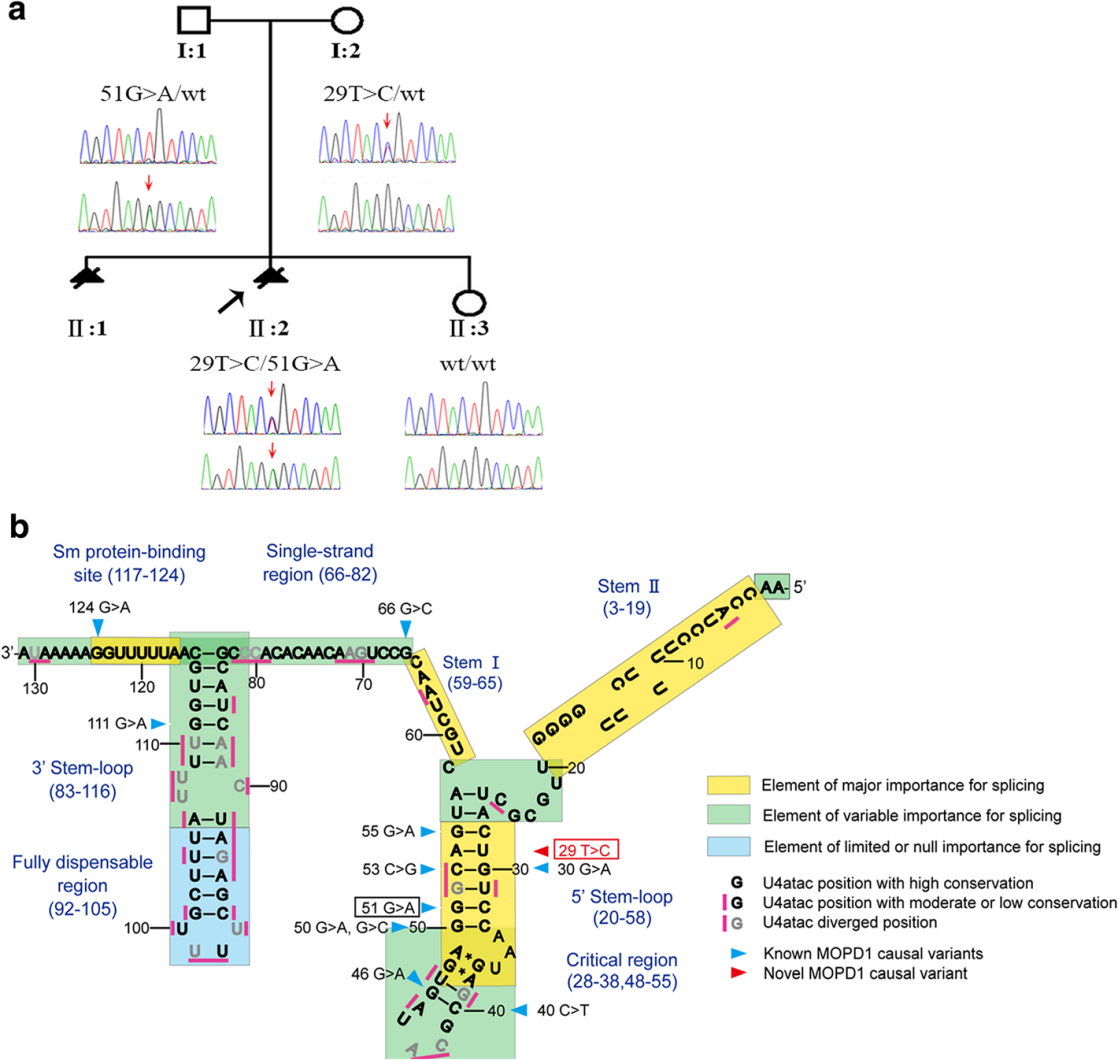
Identification of the genetic cause of severe microcephaly in a Chinese family. **a** Family pedigree. Filled triangles with oblique lines indicate the two successive foetuses affected with severe microcephaly and terminated by therapeutic abortion. Arrow indicates the proband. Open symbols indicate clinically unaffected family members. Genotypes with respect to the *RNU4ATAC* gene are also provided where it was possible to determine them. wt, wild-type. **b** U4atac snRNA secondary structure elements, evolutionary conservation status of each nucleotide position and MOPD1-causative SNVs (adapted from [46]). The novel variant found in the present study, 29T>C, is highlighted in red and boxed. For a detailed description of the structure and function of U4atac, see Merico et al. [46] and references therein

**Fig. 2:**
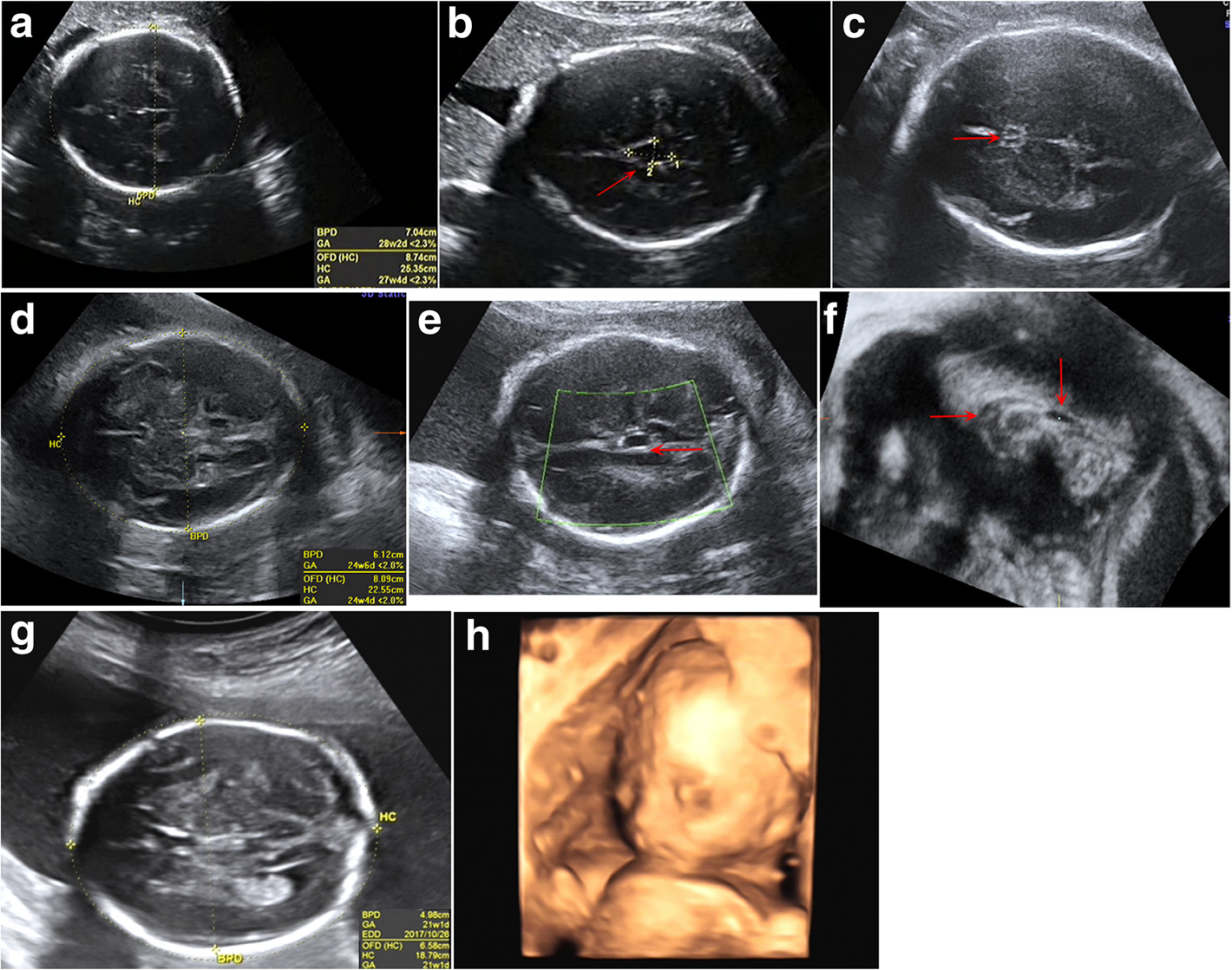
Ultrasound images of the three foetuses. **a** Biparietal diameter (BPD) and head circumference (HC) of foetus II:1 measured at 35 GW indicating severe microcephaly. **b** Cross-section plane of the skull displaying arachnoid cysts (arrow) in foetus II:1. **c**–**f** Ultrasound images of foetus II:2: BPD and HC measured at 29 GW indicating severe microcephaly (**d**); cross-section and three-dimensional sagittal plane of the skull showing the absence of the septum pellucidum cavity (arrow; **c**), presence of intracranial cyst (arrows; **e**, **f**) and agenesis of corpus callosum (arrow; **f**). **g**, **h** Ultrasound images of the healthy foetus II:3: BPD and HC (measured at 21 GW) shown in cross-section plane (**g**) and three-dimensional rebuilt imaging for foetus face (**h**)

**Table 1:** Clinical data of the two affected foetuses

Case	Foetus 1 (II:1)			Foetus 2 (II:2)		
Growth (GW)	24	33	36	24	26	29
BPD (SD)	− 6.0	− 6.0	− 6.1	− 4.4	− 5.6	− 6.0
HC (SD)	− 4.0	− 5.6	− 7.5	− 3.7	− 4.2	− 5.5
FL (SD)	− 2.0	− 1.6	− 2.0	0	− 1.0	0
HL (SD)	− 1.5	/	− 1.0	0	0	0
AC (SD)	/	− 2.4	− 3.8	− 0.7	− 1.0	− 1.4
Weight	/			1.047 ± 0.153 kg (29 GW, < 50 centile)		
Brain anomalies						
Hypogenesis or agenesis of corpus callosum	–			–		
Absence of septum pellucidum cavity	–			+		
Intracranial cysts	+			+		
Small vermis	–			–		
Skeletal anomalies						
Short limb	+ (slightly short)			–		
Flexion contractures	–			–		
Cardiac abnormalities	–			–		
Skin and skin appendage	/			/		

*BPD* biparietal diameter, *HC* head circumference, *FL* femur length, *HL* humerus length, *AC* abdomen circumference, *GW* gestational week, *SD* standard deviation, / not evaluable, − negative, + positive

The parents were of North Chinese origin, healthy and nonconsanguineous. Exposure to known causative environmental factors during pregnancy was neither reported nor identified. Taken together with the remarkably similar clinical phenotypes in the two affected foetuses (Fig. 2; Table 1), a genetic cause was considered to be likely. An extensive molecular genetic analysis was therefore performed on foetus II:2.

### Extensive karyotyping and chromosomal microarray analysis failed to identify any chromosomal abnormality or pathogenic copy number variations in II:2

We first performed standard G-banding karyotyping using cord blood cells from II:2 (taken at 29 GW), but no chromosomal abnormalities were found. In the meantime, we also performed chromosomal microarray analysis using genomic DNA prepared from the cord blood cells taken from II:2. No pathogenic copy number variants were identified by reference to data available in OMIM [11], DGV [12] and DECIPHER [13].

### WES also failed to reveal a genetic cause of the microcephaly in II:2

We further employed WES to search for putative causal variants in an unbiased and hypothesis-free manner. The resulting single-nucleotide variants (SNVs) and small insertions or deletions (indels) were subjected to the following prioritizations: (i) variants that cause non-synonymous, frameshift and in-frame changes and variants that occurred at splice sites; (ii) variants with a minor allele frequency of less than 5% according to either the 1000 Genomes Project [14] or the ESP5400 data of the National Heart, Lung, and Blood Institute GO Exome Sequencing Project [15]; (iii) in case of missense variants, those predicted to be deleterious using the programs of PolyPhen-2 [16], SIFT [17] and Mutation Taster [18] and (iv) variants occurring in known microcephaly-causing or microcephaly-associated genes as well as in candidate genes selected on the basis of known biological, physiological or functional relevance to microcephaly. However, no variants survived this process of prioritisation.

### Targeted sequencing of the noncoding *RNU4ATAC* gene identified causal variants in II:2

After failing to detect any pathogenic lesion by karyotyping, chromosomal microarray analysis and WES, we began to consider the potential involvement of noncoding RNA genes in the aetiology of microcephaly. An extensive literature research resulted in the recognition of two such genes. The first was the miR-17-92a-1 cluster host gene (*MIR17HG*; OMIM #609415). Large-scale copy number variants that serve either to delete or duplicate the entire *MIR17HG* locus cause Feingold syndrome 2 (OMIM #614326), a rare autosomal dominant disorder characterised by variable combinations of microcephaly, limb malformations, oesophageal and duodenal atresias and learning disability [19–26]. Although the disease entity under study here is most consistent with a model of autosomal recessive inheritance, we nevertheless revisited our chromosomal microarray analysis data and confirmed the absence of large deletions or duplications involving the *MIR17HG* locus.

The second noncoding gene emerging from our literature search was *RNU4ATAC*, in which homozygous or compound heterozygous variants have been reported to cause microcephalic osteodysplastic primordial dwarfism type-1 (MOPD1; OMIM #210710) in two simultaneous papers in 2011 [27, 28]. MOPD1, also known as Taybi-Linder syndrome [29], is a severe autosomal recessive disease characterised by dwarfism, microcephaly and neurologic abnormalities; patients usually die within the first year of life [30]. *RNU4ATAC* is located on chromosome 2q14.2 and encodes the highly conserved, 130-bp small nuclear RNA (snRNA) U4atac (RefSeq NR_023343.1). U4atac is a component of the minor spliceosome that is responsible for the correct splicing of the U12-dependent class of introns [31–35]. To date, a total of 12 *RNU4ATAC* variants including 11 SNVs (i.e. 30G>A, 40C>T, 46G>A, 50G>A, 50G>C, 51G>A, 53C>G, 55G>A, 66G>C, 111G>A and 124G>A) and 1 duplication variant (i.e. 16_100dup) have been reported to be causative for MOPD1 [27, 28, 36–41]. Six of the 11 SNVs (i.e. 30G>A, 50G>A, 50G>C, 51G>A, 53C>G and 55G>A) occurred within the critical canonical stem region (i.e. 28–33, 50–55) of a functionally indispensable element of U4atac, the 5′ stem-loop structure (Fig. 1b). In what follows, we shall term this critical canonical stem region the mutational hotspot region for MOPD1-causative variants.

We, therefore, speculated that variants in the *RNU4ATAC* gene, which would not have been detected by WES, might underlie the severe microcephaly in this family. Subsequent targeted testing of the *RNU4ATAC* gene by Sanger sequencing identified compound heterozygous variants, 29T>C (rs779143800) and 51G>A (rs188343279), in II:2. Carrier analysis confirmed that the two variants had been inherited from the mother and father, respectively (Fig. 1a). Although it was suspected that the affected foetus II:1 had also inherited these two variants, this could not be confirmed due to the non-availability of genetic material.

**Fig. 3:**
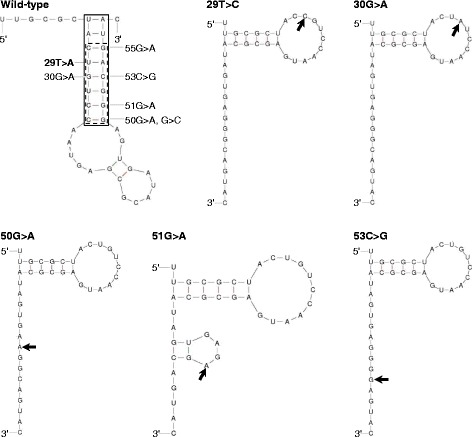
Predicted effects of 29T>C, 30G>A, 50G>A, 51G>A and 53C>G on the 5′ stem-loop structure of U4atac. Secondary structures of the wild-type and corresponding mutant sequences between positions 20 and 58 of U4atac (see Fig. 2b) were predicted by Mfold under default parameters, with the lowest energy structures being shown. In the wild-type panel, the solid-boxed area indicates the canonical stem of the 5′ stem-loop structure in accordance with [53, 54]; the dotted-boxed area indicates the mutational hotspot region for MOPD1-causative variants; all six known MOPD1-causative SNVs as well as the newly found 29T>C variant occurring within the mutational hotspot region are also indicated. In the mutant panels, the respective variants are indicated by arrows

51G>A was among the first described MOPD1-causative variants [27, 28] and represents the most common MOPD1-causative variant so far reported. By contrast, 29T>C has not been previously reported in MOPD1 patients. It has however been reported at a very low frequency in normal populations; thus, it is present in heterozygous form in two individuals in the Genome Aggregation Database [42], corresponding to an allele frequency of 1.6 × 10^− 5^. Further, in common with all 11 previously reported MOPD1-causative *RNU4ATAC* SNVs, 29T>C affected one of the evolutionarily highly conserved positions of U4atac (Fig. 1b). Furthermore, and most importantly, 29T is located within the mutational hotspot region for MOPD1-causative variants (Fig. 1b). In this latter regard, our current understanding of the pathogenetic mechanism underlying the six known MOPD1-causing *RNU4ATAC* SNVs occurring within the mutational hotspot is that they abrogate U4atac snRNA function by disrupting the 5′ stem-loop structure [27, 28, 41]. Accordingly, we compared the potential effect of 29T>C on the 5′ stem-loop structure of U4atac with those of the aforementioned six known MOPD1-causative SNVs. To this end, wild-type and the seven mutant sequences spanning positions 20 to 58 (i.e. the sequence forming the 5′ stem-loop structure; Fig. 1b) of U4atac were separately subjected to Mfold analysis [43] under default conditions. All seven SNVs were predicted to significantly affect the 5′ stem-loop structure of U4atac as compared with the wild-type. In particular, 29T>C was predicted to alter the secondary structure in the same way as the pathogenic nucleotide substitutions 30G>A, 50G>A and 53C>G; it was also predicted to alter the secondary structure in a similar way to the most common pathogenic 51G>A variant (Fig. 3). These observations, taken together, strongly suggest that 29T>C constitutes a novel causative variant for MOPD1.

### Prenatal diagnosis of the third pregnancy

Prenatal diagnosis was performed on the third foetus (II:3) (Fig. 1a). Genomic DNA was prepared from amniotic fluid cells taken by ultrasound-mediated amniocentesis at 16 GW. However, neither the *RNU4ATAC* 51G>A variant nor the 29T>C variant was detected. Normal foetal growth was confirmed by continual ultrasound monitoring during the whole period of pregnancy (Fig. 2g, h). The third foetus was born healthy after 40 GW.

## Discussion

In this study, we relate our experience of how the genetic cause was finally identified in a Chinese family presenting with two successive foetuses with severe microcephaly. In brief, negative findings from karyotyping, chromosomal microarray analysis and WES in foetus II:2 prompted us to consider the potential role of noncoding genes in causing microcephaly in the family. Consequently, targeted sequencing of the noncoding *RNU4ATAC* gene resulted in the identification of compound heterozygous variants, one being the most frequently reported MOPD1-causative 51G>A, the other being a novel 29T>A variant. Based upon the four lines of evidence, namely allele frequency in normal populations, evolutionary conservation, occurrence within a known mutational hotspot for MOPD1-causative variants and predicted effect on the 5′ stem-loop structure, we were able to conclude with confidence that the newly found 29T>A variant represents a new causative variant for MOPD1. Here, we should like to make two additional points. First, in the context of in silico analysis, many algorithms have been designed to predict the functional consequences of intronic or missense variants found in protein-coding genes. However, these tools are inappropriate for use with the *RNU4ATAC* variants discussed here, whose functional consequences depend upon their potential effect on RNA secondary structure. Currently, Mfold analysis is the gold standard for performing RNA secondary structure predictions. Second, stringent standards and guidelines have been proposed for investigating the causality of sequence variants in human genetic disease [44, 45]. Apart from the aforementioned four lines of evidence supporting causality of the detected compound heterozygous *RNU4ATAC* variants, we would like to add a new consideration. The genomic structure of *RNU4ATAC* is very simple, comprising only 130 nucleotides. All the so far reported MOPD1-causative *RNU4ATAC* variants were invariably located within the 130 nucleotides.

Most previous studies have reported homozygous or compound heterozygous *RNU4ATAC* variants in patients with a diagnosis, or suggestive diagnosis, of MOPD1 [27, 28, 36–40]. Only very recently have *RNU4ATAC* variants been described in foetuses [41]; all four foetuses (two of whom were twins) had severe microcephaly together with some other brain and skeletal abnormalities including corpus callosum agenesis, short limb, brachydactyly and ossification delay, suggestive of a diagnosis of MOPD1. By contrast, the two foetuses in the family under study here showed only severe microcephaly and corpus callosum agenesis. The identification of compound heterozygous *RNU4ATAC* variants in II:2, therefore, provided a definite diagnosis of the disease that could not otherwise have been made merely on the basis of clinical findings.

In a more general context, our study adds to the increasing appreciation that variants in noncoding RNA genes are an underestimated cause of human inherited disease. Here, we further emphasise this point by citing a recent finding concerning the *RNU4ATAC* gene. Compound heterozygous *RNU4ATAC* variants have also been reported to cause Roifman syndrome (OMIM #300258) [10, 46], a rare congenital association of antibody deficiency, spondyloepiphyseal chondro-osseous dysplasia, retinal dystrophy, poor pre- and postnatal growth and cognitive delay, which is phenotypically quite different from MOPD1. It should be noted that Roifman syndrome-causative compound heterozygous *RNU4ATAC* variants comprise one variant that is located within MOPD1-implicated structural elements and one variant that is located outside of MOPD1-implicated structural elements [10, 46].

## Conclusions

In a general context, our findings highlight one key limitation of WES, namely that it fails to detect disease causative variants within noncoding RNA genes. This provides support for a role for whole-genome sequencing as a first-tier genetic test in paediatric medicine [9]. This is also the first report of MOPD1-causative *RNU4ATAC* variants in the Chinese population and the first report of prenatal diagnosis and genetic counselling provided for a subsequent pregnancy once *RNU4ATAC* variants had been identified as a cause of MOPD1. Finally, the identification of a novel *RNU4ATAC* variant within the mutational hotspot for MOPD1-causative variants further strengthens the critical role of the 5′ stem-loop structure of U4atac in health and disease.

## Methods

### Karyotyping and chromosomal microarray analysis

Standard G-banding karyotyping was performed. The array experiments were performed using the high-resolution Affymetrix CytoScan HD microarray (Affymetrix Inc., Santa Clara, CA) in accordance with the manufacturer’s protocols. The results were analysed using the Chromosome Analysis Suite software version 1.2.2; the reporting threshold of the copy number was set at 10 kb, with marker count at ≥ 50, as previously reported [47].

### WES

Genomic DNA was fragmented randomly and then purified by means of the magnetic particle method. Sequences were captured by Agilent SureSelect version 4 (Agilent Technologies, Santa Clara, CA) according to the manufacturer’s protocols. The DNA libraries, after enrichment and purification, were sequenced on the NextSeq500 sequencer according to the manufacturer’s instructions (Illumina, San Diego). The sequencing reads were aligned to GRCh37.p10 using Burrows-Wheeler Aligner software (version 0.59) [48]. Local realignment and base quality recalibration of the Burrows-Wheeler aligned reads were then performed using the GATK IndelRealigner [49] and GATK BaseRecalibrator [50], respectively. SNVs and small indels were identified by the GATK UnifiedGenotyper [51]. Variants were annotated using the Consensus Coding Sequences Database at the National Centre for Biotechnology Information [52].

### Targeted sequencing of the *RNU4ATAC* gene

Primer sequences and PCR conditions are available upon request.

### RNA secondary structure prediction

This was performed by means of Mfold analysis under default conditions [43].

## Abbreviations

BPD: Biparietal diameter
GW: Gestational weeks
HC: Head circumference
indels: Insertions or deletions
MOPD1: Microcephalic osteodysplastic primordial dwarfism type 1
snRNA: Small nuclear RNA
SNVs: Single-nucleotide variants
WES: Whole-exome sequencing
